# Circular RNA expression profiles and features in NAFLD mice: a study using RNA-seq data

**DOI:** 10.1186/s12967-020-02637-w

**Published:** 2020-12-11

**Authors:** Xinlu Yuan, Jianjun Diao, Anqing Du, Song Wen, Ligang Zhou, Yangbin Pan

**Affiliations:** 1 Department of Endocrinology and Metabolic Diseases, Shanghai Pudong Hospital, Fudan University Pudong Medical Center, 2800 Gongwei Road, Pudong, Shanghai, 201399 China; 2 Department of Emergency, Shanghai Pudong Hospital, Fudan University Pudong Medical Center, 2800 Gongwei Road, Pudong, Shanghai, 201399 China; 3 Department of Stomatology, Shanghai Pudong Hospital, Fudan University Pudong Medical Center, 2800 Gongwei Road, Pudong, Shanghai, 201399 China; 4 Department of Nephrology, Shanghai Pudong Hospital, Fudan University Pudong Medical Center, 2800 Gongwei Road, Pudong, Shanghai, 201399 China

**Keywords:** Nonalcoholic fatty liver disease; circular RNA, Expression profile, Tissue specificity, Regulatory network

## Abstract

**Background:**

Nonalcoholic fatty liver disease (NAFLD) is primarily characterized by the hepatic cholesterol accumulation. Circular RNA (circRNA), one of noncoding RNA, involves in many liver diseases progression. However, no recent studies on circRNA expression profiles in NAFLD have been reported previously.

**Methods:**

A NAFLD mouse model was constructed by providing high-fat diet (HFD) for 32 weeks. The circRNAs expression profile in normal mice and NAFLD mice were determined using high-output RNA sequencing method and bioinformatics methods, while the differentially expressed circRNAs were confirmed using Sanger sequencing and qRT-PCR. The circRNA-miRNA network was also predicted. The biological functions of circRNAs were annotated by Gene Ontology (GO) and Kyoto Encyclopedia of Genes and Genomes (KEGG).

**Results:**

The results demonstrated the successful construction of NAFLD mice model by immunohistology and serology assay. In total, 93 dysregulated circRNAs were observed, including 57 upregulated circRNAs and 36 downregulated circRNAs, in the NAFLD group. The circRNA-miRNA network revealed the complex interaction between circRNAs and its potential miRNA targets in NAFLD. The characteristic of tissue-specific expression in circRNA was demonstrated. The differentially expressed circRNAs with important biological function were also annotated using GO and KEGG. Both *DDAH1* and *VAV3* genes were found to be associated with the NAFLD development.

**Conclusions:**

Taken together, this study demonstrated the circRNAs expression profile and features in NAFLD, which may provide potential biological markers for the pathogenesis of NAFLD.

## Background

Nonalcoholic fatty liver disease (NAFLD) represents a common chronic liver disease in many developing and developed countries [1]. The prevalence of NAFLD is 20–40% in adult, while approximately 70–80% of occurrence in diabetic and obesity patients. NAFLD encompasses a series of clinical manifestations, such as simple hepatic steatosis and nonalcoholic steatohepatitis (NASH). NASH, an important stage in NAFLD, can even develop to fibrosis, cirrhosis and hepatocellular carcinoma (HCC) [2]. Recently, the occurrence of NAFLD has increased at an alarming rate with the rapid growth of obesity in the population. There is growing evidence that NAFLD can be caused by multiple factors, including lipid accumulation in liver, mitochondrial dysfunction, a high fat diet, insulin resistance and genetic factors [3]. Although the underlying mechanism of NAFLD has not been completely explained, recent researches have suggested to find out potential biomarkers that might be able to do early prediction and diagnosis for patients with NAFLD.

Non-coding RNA molecules consist of transfer RNA (tRNA), microRNA (miRNA), and circular RNA (circRNA), which contributed greatly to many biological processes [4]. CircRNA was firstly discovered in 1976 in the viruses, which is a closed continuous loop with the deficiency of 5′ and 3′ end, in other words, circRNA cannot be degraded by RNase R and present with high stability in cells [5]. In addition, circRNA can also inhibit the miRNAs function which was found to be related to the progression and pathogenesis of chronic liver diseases [6]. The expression of circRNA is identified as extremely low in cells, however, the rapid advance of high-throughput sequencing methods has led to the high expression level of this molecule [7]. Numerous studies have revealed that the majority of circRNAs can interact with miRNA to regulate the target gene expression, that is, circRNA may be a potential biomarker and therapeutic target of NAFLD [8, 9]. Therefore, the scientific community's awareness of circRNAs has been raised due to its unique characteristics. Recently, the circRNAs expression profiles have been reported to mediate neurological diseases, diabetes and lung metastasis. A previous study conducted by Qu et al. [10], have suggested a small number of dysregulated circRNAs in the NASH mice fed with methionine/choline deficient diet, and constructed a complex circRNA-miRNA pathway interaction. However, at this stage, not much information on the circRNAs expression profiles in hepatic tissues of NAFLD have been reported.

This study demonstrated the differentially expressed circRNAs in an NAFLD mouse model. This study aimed to find out the potential roles of circRNAs in the progression and pathogenesis of NAFLD. The RNA sequencing, RT-qPCR validation and multiple bioinformatics technologies were used to investigate the differentially expressed circRNAs, followed by identify a circRNA-miRNA interaction network that is closely associated with the progression of NAFLD.

## Materials and methods

### Establishment of NAFLD mice model

Totally 20 male mice (8 weeks old, weighting 16–20 g) were obtained from Changzhou Cavens Experimental Animal Co., Ltd. All animals were kept in plastic cages at a constant temperature of 25 ℃, humidity of 40% and 12 h light/12 h dark cycle. The mice also had free food and water access during the experiment. In addition, we randomly divided the mice into two groups and provided with different diets for 32 weeks: control group fed with standard chow (n = 10) and NAFLD group fed with high-fat diet (HFD) (n = 10). The HFD was purchased from the Research Diets, Inc., New Brunswick, NJ (60% kcal fat; D12492). The levels of blood glucose were measured after fasting for 12 h. After that, all mice were anesthetized and sacrificed to collect blood for biochemical analysis, while the livers were harvested and weighted immediately. Serum and liver samples were stored in liquid nitrogen at − 80 ℃ for subsequent experiments. A small pieces of hepatic tissues were fixed in 4% paraformaldehyde (PFA) > 48 h at 4 ℃ for histological analysis. This study was approved by the Animal Research Ethical Committee of the Fudan University Pudong Medical Center. All experimental procedures followed the Guidelines for the Care and Use of Laboratory Animals of the National Institute of Health in China.

### Biochemical analysis

Glucose tolerance test (GTT) and insulin tolerance test (ITT) were determined after fasting for 12 h. The 1.5 g kg^−1^ glucose was injected intraperitoneally to conduct GTT. In contrast, 0.5 IU kg^−1^ insulin was injected for ITT. After injection, the blood glucose level was determined using OneTouch Ultra Glucose Test Strips (LifeScan Inc., Milpitas, CA) at different timelines, including 0, 30, 60, 90, and 120 min.

Plasma triglyceride (TG) was measured using Triglyceride Kit (Wako Diagnostics, Richmond, VA), while the Cholesterol Assay Kit (BioVision, Irvine, CA) was used to determine the level of total cholesterol (TC). Plasma insulin was also measured with a MILLIPLEX(®) MAP Mouse Metabolic Magnetic Bead Panel kit following the manufacturer’s instructions. To determine the hepatic TG and TC levels in control and NAFLD group, hepatic tissues were rinsed with phosphatebuffered saline (PBS) and collected in isopropanol. The homogenate was generated after centrifugation at 12 000 × *g* for 15 min and then incubated at 4 °C. The supernatants were used for further analyses.

### Histological analysis

The hepatic tissues from each mouse were fixed in 4% paraformaldehyde, followed by dehydrated in grades of alcohol, and embedded in paraffin wax (Sakura, Tokyo, Japan). The sections with 5 μm thickness were stained with hematoxylin and eosin (H&E). The slices were also stained with Oil Red O (ORO) to analyze the accumulation of hepatic lipid. A light microscope was used to observe histological features of liver tissues under × 200 (Olympus, Tokyo, Japan).

### RNA isolation and quality control

Total RNA was extracted and purified using TRIzol reagent (Invitrogen, CA, USA) and RNeasy Mini kit (Qiagen). The RNA concentration was assessed using a spectrophotometer (NanoDrop ND-1000, Thermo Scientific), while the RNA integrity was measured using electrophoresis.

### The analysis of CircRNA sequencing

RNA sequencing was applied using RNA samples from each group, while the RNase R was treated to remove linear RNAs from isolated RNA. The amplified circRNAs were reverse transcribed into cDNA using random primers following the manufacturer’s instructions. The cDNA was then synthesized and purified using the QiaQuick PCR extraction kit (Qiagen). The cDNA library was finally prepared according to illumine TruSeq library preparation instruction. CircRNA sequencing was conducted on an Illumina HiSeqTM 4000 (Illumina, CA, U.S.A).

### Reverse transcription-quantitative polymerase chain-reaction (RT-qPCR)

RT-qPCR was carried out not only to validate the results of RNA sequencing, but also to detect the expression of DDAH1 and VAV3. The isolated RNAs from liver tissues were reversely transcribed into cDNA using the RT-PCR kits (Takara) in accordance with the manufacture’s protocol, followed by amplifications using a SYBR Premix Ex Taq kit (Takara). The thermal conditions are as follows: 95 ℃ for 5 min, followed by 40 cycles of 10 s at 95 ℃, 60 ℃ for 30 s and 72 ℃ for 30 s. β‑actin was used as the internal reference. PCR bands were gel-purified and sequenced using Sanger method. The algorithm 2-ΔΔCq method was applied to normalize the relative gene expressions to β‑actin. The primer sequences used for RT-qPCR are listed in Table 1 and Additional file 4: Table S3.

**Table 1:** Primers sequences of selected circRNAs and internal reference for reverse transcription-quantitative polymerase chain-reaction (RT-qPCR) validation

Forward Sequence (5′-3′)	Name	Reverse Sequence (5′-3′)	Length
5′-GCTTCTAGGCGGACTGTTAC-3′	β-actin-R1	5′-CCATGCCAATGTTGTCTCTT-3′	100 bp
5′-GCTCCTGGGAAGGTGACATC-3′	mmu_circ_0015959-R2	5′-CCTGAGGAGTTTCCTGGAAG-3′	144 bp
5′-TGTGGCCGATTCTTTGCATT-3′	mmu_circ_0010514-R2	5′-CATCATGTCAACCTTGAGGG-3′	109 bp
5′-CTTAGATCAGCCGTGTTGTG-3′	mmu_circ_0004580-R2	5′-ACAGTCCCATTAAGCCTTGC-3′	144 bp
5′-ATAGATGGCTGGGGCTTTGG-3′	chr7_82671604_82674582-R1	5′-GCGAACCGCTGTTGATACTT-3′	100 bp
5′-GCGACTCAGACACAGATCCA-3′	chr17_66049785_66053091-R1	5′-ACTGCTGTCACTGTCAGAAT-3′	126 bp
5′-AACACTCTGCACGGGTCAAG-3′	chr4_46734057_46749582-R1	5′-TCCGAAGCTGCTCTAGAATG-3′	203 bp
5′-GAACGTCCTATATCATTAGGG-3′	chr11_94036599_94048558-R4	5′-TTCAAGTCTGCTGACTTCAG-3′	161 bp
5′-CATGAACTGCAGGGCTGAAC-3′	chr5_118593332_118593570-R1	5′-GCAGACAGCGGATGAAACTT-3′	134 bp

### RNA sequencing and bioinformatics analysis

The raw data with low-quality reads were filtered out using Qubit3.0, while the remaining reads were mapped to the mouse genome (GRCm38) using Bowtie2 v2.2.8. The reference genome was established by software TopHat2 v2.1.1 (25, 26). The remaining reads after alignment were subjected to CIRCexplore, MapSplice and CircRNA_finder software for circRNA identification. The chromosome distribution of the identified circRNAs were evaluated. The circRNAs were categorized as significantly differentially expressed using edgeR package (fold change ≥ 1.5 and *p*-value < 0.05). The differentially expressed circRNAs were selected using volcano plotting. Gene ontology (GO) was applied to annotate meaningful gene products, which contains three categories of biological function, namely biological process (BP), cellular components (CC) and molecular function (MF). In contrast, Kyoto Encyclopedia of Genes and Genomes (KEGG) was utilized to identify the target genes in biological pathways.

Once the differentially expressed circRNAs are identified, StarBase v2.0 software was used to predict the target miRNAs. Mireap (https://sourceforge.net/projects/mireap/), Miranda v3.3a, (http://miranda.org.uk/) and TargetScan v7.0, (http://www.targetscan.org) databases were applied to predict the novel circRNAs. After that, the circRNA-miRNA network was visualized using Cytoscape software. To improve the reliability of our prediction, the match score was set as > 140 and the minimum free energy < − 20.

### Statistical analysis

All data are expressed as mean ± standard deviation. An independent sample t-test was carried out to determine differences between groups using SPSS (version 23.0; IBM Corp., Armonk, NY, USA). Statistical significance was set at *p* < 0.05.

## Results

### The construction of the NAFLD mouse model

**Fig. 1:**
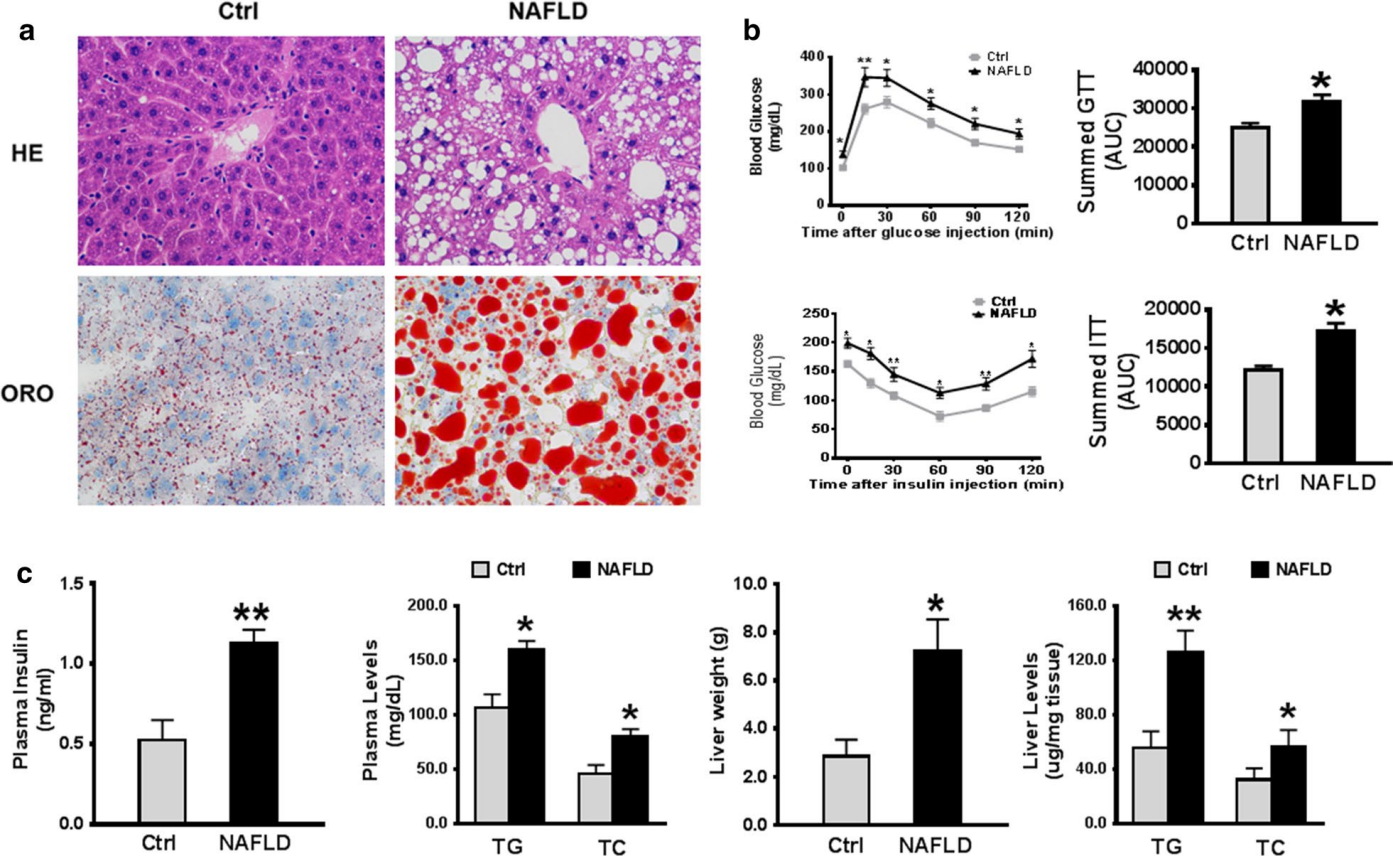
**a** The immunohistochemistry of liver tissues from control and NAFLD group using H&E and Oil Red O staining, respectively (magnification 200x). **b** The changes of blood glucose after glucose or insulin injection, as well as the results of GTT and ITT in control and NAFLD group. **c** Plasma insulin, plasma TG, plasma TC, the weight of the liver, and the plasma TG and TC content in control and NAFLD group. *p < 0.05, **p < 0.01

NAFLD mouse model was successfully constructed after 32 weeks HFD, as evidenced by significant fluctuations in serum levels and the presentation of hepatic tissues. The results of H&E staining demonstrated the distinct histopathological features of NAFLD. As shown in Fig. 1, sections from NAFLD model group had disordered liver lobules, excessive fat-containing vacuoles in cytoplasm, and cellular swelling. The HFD-induced mice demonstrated the accumulation of fat in the liver under the ORO staining (Fig. 1a). Compared to controls, the blood glucose levels in the NAFLD group were significantly higher after glucose injection, especially at time period of 30 min. Both NAFLD and control groups had declined blood glucose after insulin injection at different timeframe, but the level of blood glucose in NAFLD group was still higher than the control group (Fig. 1b). Both summed GTT and ITT of NAFLD group were significantly raised (*p* < 0.05). Additionally, the levels of plasma TC, TG, plasma insulin and liver weight were dramatically elevated compared to the controls (*p* < 0.05). We also determined the levels of TG and TC within the livers. Our data reflected the hepatic TC and TC levels were noticeably elevated in the NAFLD group (Fig. 1c). These data in serum-based biomarkers and hepatic tissues indicated the successfully construction of the NAFLD mice model.

### Expression profile of circRNAs in NAFLD mice

**Fig. 2:**
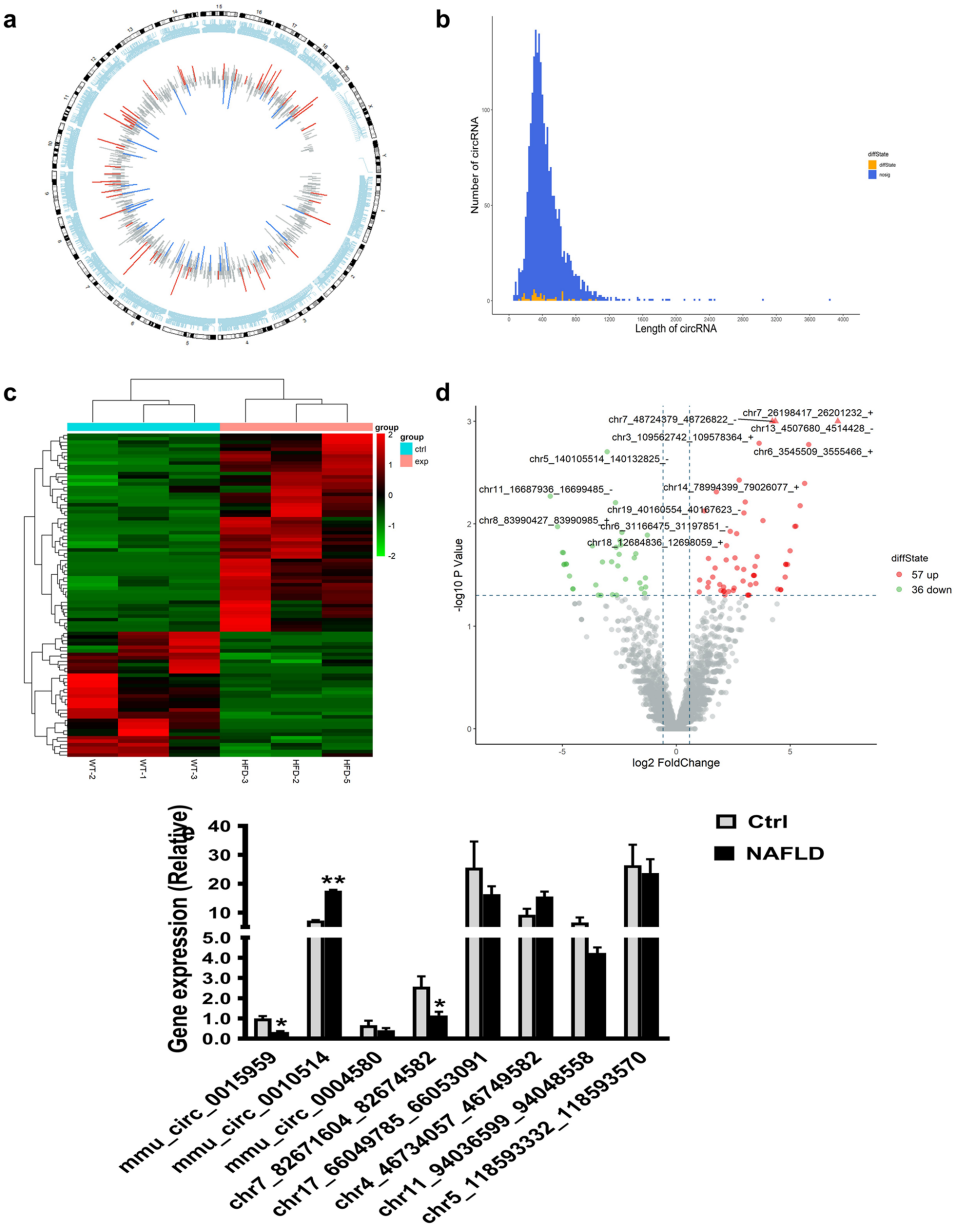
Bioinformatics analysis of differentially expressed circRNA in NAFLD and normal mice. **a** The distribution of differentially expressed circRNA on mouse chromosome. The length of lines indicates the relative size of fold-change; Red: up-regulated circRNAs; Green: down-regulated circRNAs. **b** The distribution of circRNA length. Blue: not significantly expressed circRNAs; Orange: the significantly expressed circRNAs. **c** Heat maps reflected different circRNA expression profiles between NAFLD group and control group. **d** Volcano plots comparing differentially expressed circRNA in NAFLD mice with control. Red: up-regulated circRNAs; Green: down-regulated circRNAs. **e** The expression levels of eight random selected circRNAs after RT-qPCR analysis. *p < 0.05, **p < 0.01

To identify the differentially expressed circRNAs between the NAFLD group and control group, circRNA sequencing was conducted in each group. A circular diagram represented the types and distributions of identified circRNAs on the chromosome (Fig. 2a). The length of lines indicates the fold-change. As shown in Fig. 2b, the distribution of circRNAs length was showed with the significantly differentially expressed circRNA in orange and in-differentially expressed circRNA in blue. In addition, hierarchical clustering and volcano plot exhibited the significant differentially expressed circRNAs in NAFLD and controls (Fig. 2c, d; FC ≥ 1.5, *p* < 0.05). Hence, 93 circRNAs of NAFLD mice were considered as dysregulated, in which 57 of upregulated circRNAs and 36 of downregulated circRNAs (Additional file 1: Table S1). Overall, the above results suggested the different circRNA expression patterns in NAFLD livers, compared to the control group.

Additionally, GO terms and KEGG pathway were carried out to interpret the biological functions of 93 differentially expressed circRNAs. As shown in Fig. 4, the top 10 highly enriched GO biological process (BP) and 5 highly enriched GO cellular component (CC) and molecular function (MF) were demonstrated (Fig. 4a). The most enriched GO terms in BP was ‘fatty acid metabolic process’ (*p* < 0.0001), that in CC were ‘endosome membrane’ and ‘endosome part’, and that in MF were ‘Ras GTPase binding’ and ‘small GTPase binding’. KEGG pathway analysis was further conducted to find out the signalling pathway that the genes participate, which obtained eight significantly enriched pathways (Fig. 4b). According to the results of KEGG, ‘cAMP signalling pathway’ ranked the first among the differentially expressed circRNAs, which is considered to be associated with the attenuation of obesity in NAFLD.

### Validation of circRNA expression profiles by RT-qPCR

**Fig. 3:**
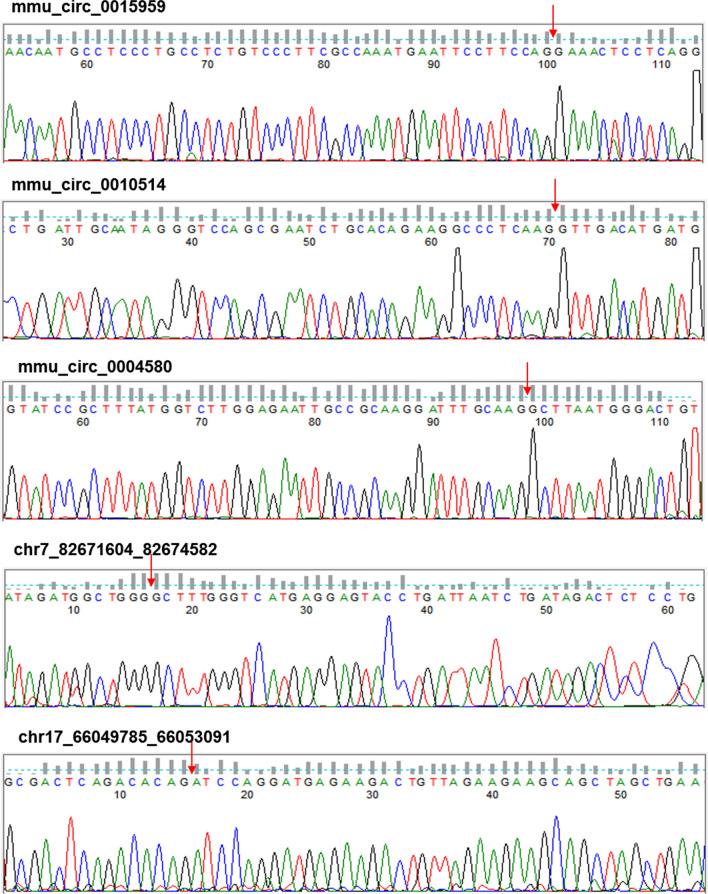

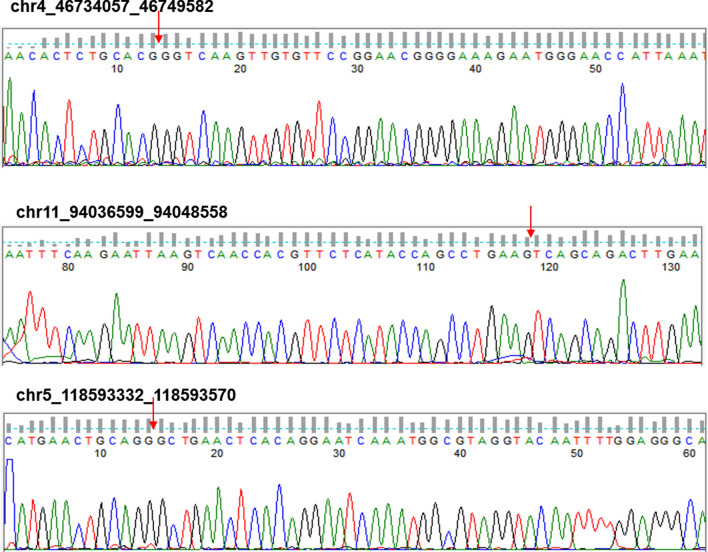
Head-to-tail splicing in the RT-qPCR product of eight random selected circRNAs

To validate the sequencing results of circRNA expression profiles, we randomly selected eight differentially expressed circRNAs for RT-qPCR, including seven upregulated circRNAs and one downregulated circRNA. Primers for circRNA with forward and reverse sequences were illustrated in Fig. 3. The results of RT-qPCR suggested that both circRNA_0010514 and chr4_46734057_46749582 were upregulated, and these findings were the same as the results obtained from the RNA-seq data. Additionally, mmu_circ_0015959, mmu_circRNA_0010514, and chr7_82671604_82674582 reached the required expression level of statistical significance (Fig. 2e). On the other hand, the rest of the circRNAs had the opposite expression in RNA sequencing and RT-qPCR analysis, with no significant differences. The expected head-to-tail junctions of eight circRNAs was checked by Sanger sequencing (Fig. 3).

Moreover, a set of eight differentially expressed circRNAs were randomly selected for another round of RT-qPCR validation, which reflected 6 out of 8 circRNAs had consistent results with the RNAseq data (Additional file 2: Fig. 1), among which the expression of chr1_82340318_82342862, chr6_37353556_37364143 and chr9_21742186_21742796 were statistically significant (*p* < 0.05). The primer sequence of each circRNA is present in Additional file 3: Table S2.

### Prediction of circRNA-miRNA network

**Fig. 4:**
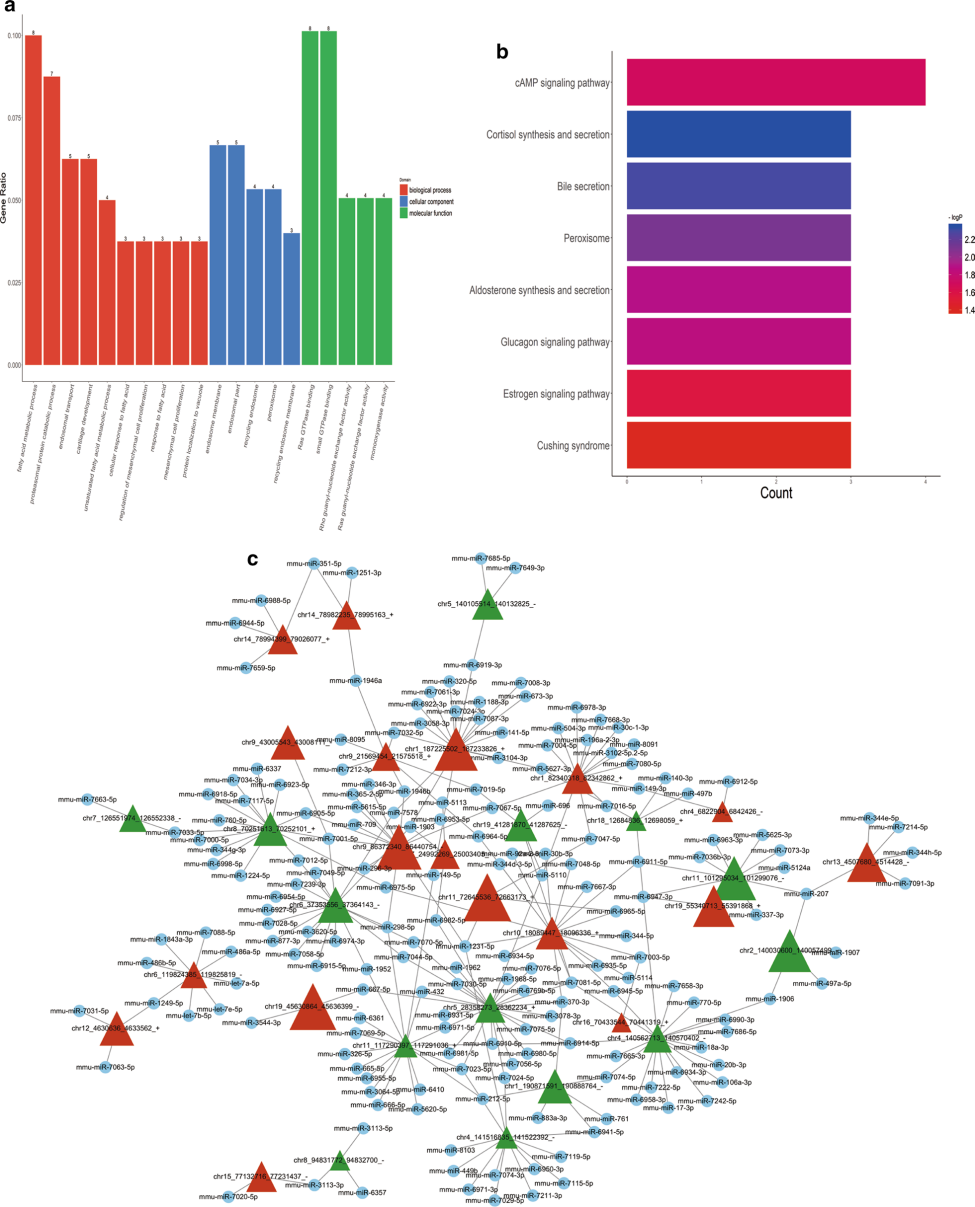
**a** GO analysis of 57 upregulated circRNAs and 36 downregulated circRNAs. *p* < 0.05. The GO terms were classified by BP, CC, and MP. GO, Gene Ontology; BP, biological process; MP, molecular function. **b** KEGG analyses of 93 differentially expressed circRNAs. The count represents the enrichment of each pathway. **c** CircRNAs-miRNAs interaction network of top 300 dysregulated circRNAs. Red triangle: upregulated circRNA; Green triangle: downregulated circRNA; Blue node: miRNA

Given that circRNAs serve a significant biological role in the miRNA target, such as the regulation of gene expression. Herein, we selected the correspond miRNAs from miRbase database, and used miRanda software to detect the interaction between the miRNA with the differentially expressed circRNAs. The higher miRNA response elements (MRE) frequency, the more circRNAs binding sites with miRNA. We therefore built and displayed a circ-RNA-miRNA network of top 300 dysregulated circRNAs using software Cytoscape to exhibit the complex interaction (Fig. 4c and Additional file 4: Table S3).

## Discussion

NAFLD is closely related with high-fat diet, obesity, insulin resistance, inflammation, and genetic factors [11]. Several studies have suggested that inflammation can contribute to the development of liver diseases, while Angulo [12] in 2002 has reported the close relationship of hepatic steatosis and obesity with NAFLD patients. A deeper understanding of the underlying mechanism of NAFLD is necessary for accurate diagnosis and new effective treatments. CircRNAs are found to be linked with many diseases, biological processes and gene expressions in earlier researches [13, 14]. Tissue-specific expression is one of the characteristics of circRNAs, which allows circRNA to inhibit the miRNAs activity, suggesting the regulatory role of circRNAs in the progression of diseases [4]. Previous studies have also indicated the close relationship between circRNAs with hepatic steatosis and NASH, and circRNAs can regulate the cancer cell growth, proliferation, migration and invasion. Guo et al. [3] have reported the dysregulation of circRNAs is related to the hepatic steatosis. To date, not many researchers have paid sufficient attention to the important role of circRNAs in NAFLD. Given the prevalence of NAFLD, further investigation of circRNAs profiles may provide insight into the pathogenic mechanism of NAFLD.

**Fig. 5:**
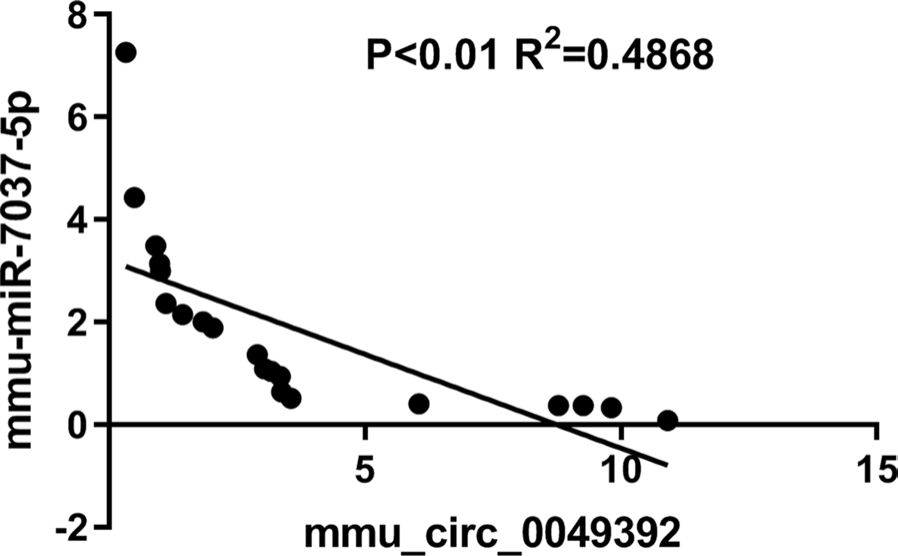
A correlation analysis of the data of mou_circ_0049392 and miR-7037-5p with serum parameter. r = 0.6977, indicating the strong negative relationship between mmu_circ_0049392 and mmu_miR-7037-5p

In our study, a long-term HFD-induced NAFLD in mouse model was firstly established, H&E and ORO staining were applied to confirm the success of NAFLD model. The serum results combined with the staining analysis further confirmed a successful simulation of NAFLD in the mice (Fig. 1). After 32 weeks, the groups of NAFLD mice exhibited disordered hepatic lobules and fat accumulation in the liver, accompanied by significantly increased plasma TG, TC levels and liver weight (*p* < 0.05). The detected circRNAs of NAFLD mouse liver tissues were distributed on all the mouse chromosomes, however, not on the Y chromosome (Fig. 2a). In this work, 93 dysregulated circRNAs with the threshold of FC ≥ 1.5 and p < 0.05 were observed based on the heatmap and volcano plotting (Fig. 2b–d), including 57 upregulated and 36 downregulated circRNAs in the NAFLD group. Following validation by RT-qPCR, most of circRNAs were differentially expressed in NAFLD group. Moreover, compared to the control group, the significantly differentially expressed circRNAs were annotated in NAFLD group, and the circRNA-miRNA network was also predicted. Different circRNAs might have various potential miRNA targets. The target miRNAs of mmu_circRNA_0049392 are miR-7037-5p and miR-6919-5p, which have been validated as a receptor of low-density lipoprotein (LDL). A correlation analysis of mmu_circ_0049392 and miR-7037-5p with serum parameter was conducted and revealed a significant negative correlation between mmu_circ_0049392 and mmu_miR-7037-5p (*p* < 0.01, |r|= 0.6977), suggesting that circRNA do function as ‘miRNA sponge’ to inhibit the activity of miRNAs, and therefore regulate the gene expression at transcription level (Fig. 5). The abnormal gene expression was found to be associated with atherosclerosis, obesity and diabetes. LDL receptor encoded by *Ldlr* gene allows the degradation of LDL [15]. A study conducted by Kelli et al., in 2017 [16] have reflected the attenuated disease development, decreased triglycerides accumulation and inflammation responses in *Ldlr*−/− mice fed with HFD. Hence, further experiments of the role of circRNA_0049392 in NAFLD is required.

**Fig. 6:**
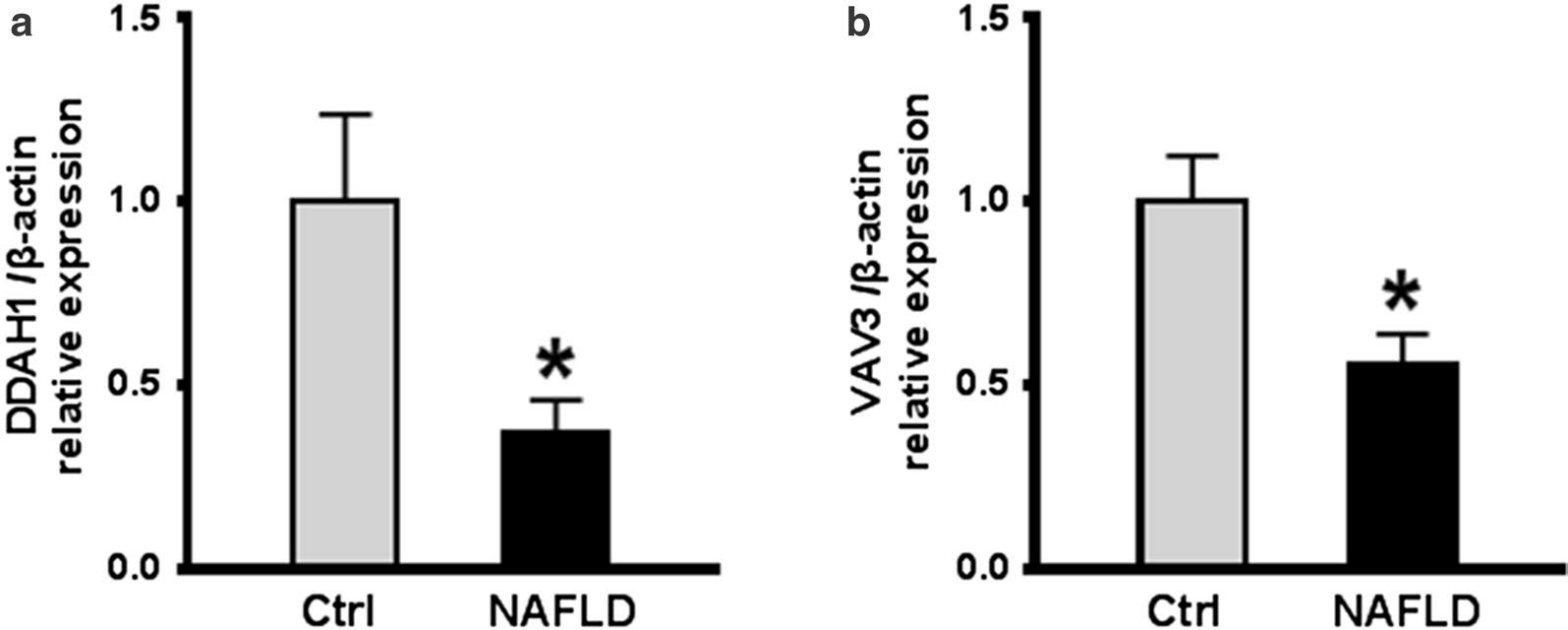
The results of *DDAH1* **a** and *VAV3* **b** expression in control and NAFLD mice using RT-qPCR. *p < 0.05

To explore the regulatory roles of cricRNAs in the pathogenesis of NAFLD, the GO terms of these genes in network were analyzed. We found that the circRNAs are enriched in the fatty acid metabolic process. In GO terms of MF and CC, these circRNAs were mostly enriched in small GTPase and endosome membrane, respectively. The results of KEGG pathway analysis indicated circRNAs were related to the signaling pathway of cAMP, and cAMP serves as a positive mediator in the attenuation of steatosis and obesity in NAFLD [17]. Additionally, *DDAH1* and *VAV3* genes were found to be associated with the development of NAFLD, while the target miRNA of circRNA chr3_145845704_145853276_ + and chr3_109562742_109578364_ + may be inhibited to regulate the DDAH1 and VAV3 expressions. Dimethylarginine Dimethylaminohydrolase 1 (DDAH1) is an enzyme that degrades Asymmetric dimethylarginine (ADMA), and is highly expressed in the liver [18], while increased ADMA levels are reported in NAFLD [19]. The ADMA-DDAH1 pathway was found to have a remarkable effect on the hepatic lipogenesis of HFD-induced mice, in other words, DDAH1 may protect against NAFLD via attenuating ADMA accumulation [18]. The decreased expression of DDAH1 in NAFLD mice indicated the protective effect of DDAH1 against NAFLD (Fig. 6a). Moreover, VAV3 is a Rho family GTPase guanine nucleotide exchange factor (GEF), and the activation of VAV3 in a pathway involves in the actin cytoskeletal rearrangement [20]. A previous study has also suggested the leukocytes often migrate and invade the hepatic lobules in the HFD-induced mice, while the VAV3-activated the pathway for leukocytes motility and direction sensing. The dropped VAV3 expression in model mice reflected that VAV3 played an important role in protecting mice from NAFLD (Fig. 6b). Taken together, the above findings suggested *DDAH1* and *VAV3* genes might serve as a potential biological marker of NAFLD development.

**Fig. 7:**
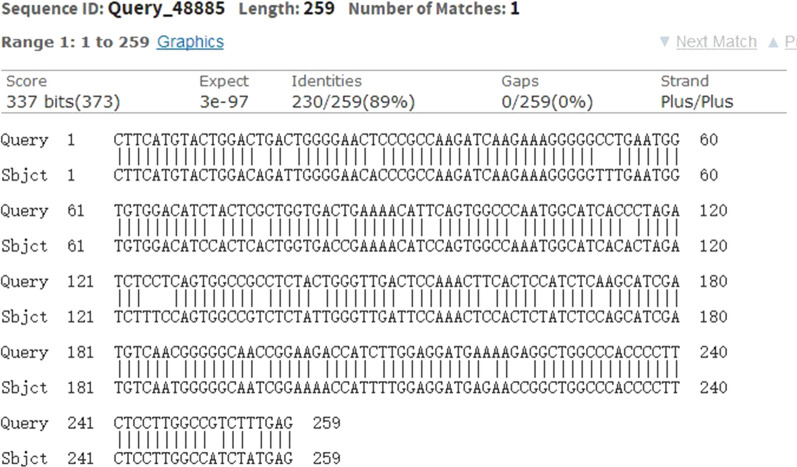
The results of the homologous sequence analysis of circRNA_0049392 comparing between mouse (sbjct 1) and human (Query 1). The mouse shared 89% sequence homology compared with its sequence in humans. CircRNA, circular RNA

As shown in Fig. 7, a homologous sequence analysis of circ_0049392 comparing between mouse and human found that mouse shared nearly 89% of sequence homology with the sequence of has_circ_0049392 in human, however, the expression profile in human NAFLD still requires further investigation. In addition, it is not possible to exclude other dysregulated circRNAs that involved in the pathogenesis of NAFLD due to the low validation rate of RNAseq results with the RT-qPCR results, and therefore future study on this issue is required to provide a promising avenue for research. Moreover, it is not applicable to detect circRNA in human liver to confirm the early diagnostic biomarker of NAFLD.

## Conclusions

In conclusion, we identified 93 dysregulated circRNAs in the NAFLD mouse liver tissues. We constructed a network to illustrate the association between differentially expressed circRNAs and their potential target miRNAs. *DDAH1* and *VAV3* genes were found to be associated with the development of NAFLD. This present study is the first one to explore the circRNAs profiles in NAFLD using RNA sequencing, RT-qPCR and bioinformatics analysis. This paper has demonstrated the significant role of circRNAs in the pathogenesis of NAFLD.

## Supplementary Information

**Additional file 1: Table S1.** The fold-change and p value of 93 differentially expressed circRNAs.

**Additional file 2: Figure S1.** The expression levels of another eight random selected circRNAs after RT-qPCR validation. *p<0.05, **p<0.01.

**Additional file 3: Table S2.** Primers sequences of randomly selected eight circRNAs and two genes for another reverse transcription-quantitative polymerase chain-reaction (RT-qPCR).

**Additional file 4: Table S3.** The circRNAs-miRNAs interaction network of top 300 dysregulated circRNAs.

## Data Availability

The datasets during and/or analyzed during the current study available from the corresponding author on reasonable request.

## Abbreviations

NAFLD: Nonalcoholic fatty liver disease
circRNAs: Circular RNAs
HFD: High-fat diet
GO: Gene ontology
KEGG: Kyoto Encyclopedia of Genes and Genomes
NASH: Nonalcoholic steatohepatitis
HCC: Hepatocellular carcinoma
tRNA: Transfer RNA
miRNA: MicroRNA
GTT: Glucose tolerance test
ITT: Insulin tolerance test
TG: Triglyceride
TC: Total cholesterol
PBS: Phosphatebuffered saline
H&E: Hematoxylin and eosin
ORO: Oil Red O
RT-qPCR: Reverse transcription-quantitative polymerase chain-reaction
BP: Biological process
CC: Cellular components
MF: Molecular function
MRE: MiRNA response elements
LDL: Low-density lipoprotein
DDAH1: Dimethylarginine Dimethylaminohydrolase 1
ADMA: Asymmetric dimethylarginine
GEF: GTPase guanine nucleotide exchange factor
