# Supplementary material for: Circular RNA expression profiles and features in NAFLD mice: a study using RNA-seq data
Source: J Transl Med. 2020 Dec 11;18:476. doi: 10.1186/s12967-020-02637-w (PMC7731504; doi:10.1186/s12967-020-02637-w)
Supplement: Supplementary file 1 — Additional file 1: Table S1. The fold-change and p value of 93 differentially expressed circRNAs. [file 12967_2020_2637_MOESM1_ESM.doc]

**Supplementary Table 1.** The fold-change and p-value of 93 differentially expressed circRNAs. Chr_Start_End_Strand: genomic location of circRNAs; Gene: host gene or derived-parent gene; FC: fold-change.

| Chr_Start_End_Strand | Gene | LogFC | FC | p-value |
| --- | --- | --- | --- | --- |
| chr7_26198417_26201232_+ | Cyp2b9 | 7.091372 | 136.369 | 3.78E-07 |
| chr6_3545509_3555466_+ | Ccdc132 | 5.808848 | 56.05799 | 0.001681 |
| chr11_72645536_72663173_+ | Ube2g1 | 5.632354 | 49.60295 | 0.004031 |
| chr19_45630864_45636399_- | Fbxw4 | 5.439792 | 43.40508 | 0.006648 |
| chr9_86372340_86440754_- | Ube2cbp | 5.246273 | 37.95645 | 0.010538 |
| chr8_84860905_84861430_+ | Farsa | 5.19815 | 36.71125 | 0.010598 |
| chr17_79650301_79651801_+ | Rmdn2 | 4.997093 | 31.93559 | 0.018347 |
| chr1_187225502_187233826_+ | Gpatch2 | 4.877244 | 29.38981 | 0.024888 |
| chr10_117777128_117777664_- | Nup107 | 4.805998 | 27.97368 | 0.024843 |
| chr17_39844471_39845587_+ | Rn45s | 4.803354 | 27.92246 | 0.024753 |
| chr3_145845704_145853276_+ | Ddah1 | 4.777638 | 27.42915 | 0.033063 |
| chr17_66049785_66053091_- | Ankrd12 | 4.58261 | 23.96089 | 0.043952 |
| chr19_55340713_55391868_+ | Vti1a | 4.58261 | 23.96089 | 0.043952 |
| chr4_46734057_46749582_- | Gabbr2 | 4.453434 | 21.90873 | 0.043199 |
| chr13_4507680_4514428_- | Akr1c20 | 4.345853 | 20.33443 | 0.000651 |
| chr7_48724379_48726822_- | not_annotated | 4.212394 | 18.53774 | 1.44E-11 |
| chr11_116160061_116163359_- | Fbf1 | 3.804994 | 13.97711 | 0.009297 |
| chr3_109562742_109578364_+ | Vav3 | 3.628544 | 12.36803 | 0.001635 |
| chr12_4630636_4633562_+ | Itsn2 | 3.545599 | 11.67701 | 0.020908 |
| chr2_158492363_158493013_+ | Ralgapb | 3.489883 | 11.23464 | 0.038088 |
| chr9_43005543_43008111_- | Arhgef12 | 3.455891 | 10.97304 | 0.025967 |
| chr9_86598718_86613638_- | Me1 | 3.43029 | 10.78004 | 0.03185 |
| chr14_21318012_21381617_+ | Adk | 3.397324 | 10.5365 | 0.031855 |
| chr7_48720133_48726822_- | not_annotated | 3.384866 | 10.44591 | 0.031836 |
| chr18_70580652_70593340_+ | Mbd2 | 3.246879 | 9.4931 | 0.03933 |
| chr11_106236994_106240099_+ | Ddx42 | 3.190855 | 9.13152 | 0.049415 |
| chr10_18089447_18096336_+ | Reps1 | 3.160559 | 8.94176 | 0.049497 |
| chr14_27438598_27442620_+ | Fam208a | 3.128321 | 8.74417 | 0.049527 |
| chrX_56643367_56655053_+ | Slc9a6 | 3.026345 | 8.147431 | 0.006126 |
| chr1_82340318_82342862_+ | Rhbdd1 | 2.9667 | 7.817458 | 0.007851 |
| chr13_3556313_3557057_+ | Gdi2 | 2.962029 | 7.79219 | 0.027827 |
| chr15_77132716_77231437_- | Rbfox2 | 2.93142 | 7.628611 | 0.036159 |
| chr14_78994399_79026077_+ | Vwa8 | 2.763754 | 6.791611 | 0.003737 |
| chr17_24992269_25003405_- | Cramp1l | 2.667349 | 6.352607 | 0.026893 |
| chr9_21569454_21575518_+ | Carm1 | 2.648812 | 6.271505 | 0.012473 |
| chr14_78982235_78995163_+ | Vwa8 | 2.577226 | 5.96791 | 0.021461 |
| chr9_72591750_72593360_+ | Rfx7 | 2.538817 | 5.811122 | 0.042328 |
| chr9_119150823_119154005_- | Acaa1b | 2.498766 | 5.652018 | 0.044669 |
| chr11_96322110_96323445_+ | Hoxb3 | 2.365105 | 5.151903 | 0.01182 |
| chr6_119824385_119825819_- | Erc1 | 2.29317 | 4.90132 | 0.045649 |
| chr16_20656542_20657609_+ | Psmd2 | 2.206931 | 4.616922 | 0.016333 |
| chr17_26083575_26084135_- | Decr2 | 2.190017 | 4.563109 | 0.022472 |
| chr11_94036599_94048558_+ | Spag9 | 2.131008 | 4.380235 | 0.049449 |
| chr5_127650682_127657138_+ | Glt1d1 | 2.097559 | 4.279846 | 0.045195 |
| chr6_141663577_141672313_+ | Slco1b2 | 2.075811 | 4.215812 | 0.04573 |
| chr18_5633203_5705243_+ | Zeb1 | 2.074471 | 4.2119 | 0.04126 |
| chr19_21678352_21684305_+ | Abhd17b | 1.950321 | 3.864604 | 0.044599 |
| chr1_121319596_121319972_- | Insig2 | 1.904678 | 3.744253 | 0.039023 |
| chr10_107045212_107053516_- | Acss3 | 1.756306 | 3.378321 | 0.004888 |
| chr10_107023653_107053516_- | Acss3 | 1.701941 | 3.253383 | 0.026998 |
| chr8_13136795_13142598_+ | Cul4a | 1.594095 | 3.019051 | 0.033025 |
| chr4_151830417_151835883_- | Camta1 | 1.41335 | 2.66355 | 0.037314 |
| chr4_6822904_6842426_- | Tox | 1.40684 | 2.651558 | 0.021723 |
| chr6_141648488_141657606_+ | Slco1b2 | 1.381055 | 2.604588 | 0.04156 |
| chr16_70433544_70441319_+ | Gbe1 | 1.232839 | 2.350291 | 0.007492 |
| chr5_118593332_118593570_+ | Med13l | 1.028389 | 2.039745 | 0.035317 |
| chr11_84193078_84195687_+ | Acaca | 1.008691 | 2.012085 | 0.046235 |
| chr18_12684836_12698059_+ | Ttc39c | -1.27978 | 0.411859 | 0.012863 |
| chr8_94831772_94832700_- | Ciapin1 | -1.34718 | 0.393059 | 0.041331 |
| chr17_64710703_64714712_+ | Man2a1 | -1.36442 | 0.38839 | 0.033634 |
| chr3_119194241_119195188_+ | Dpyd | -1.40182 | 0.378452 | 0.047478 |
| chr3_41581194_41596826_+ | Jade1 | -1.54169 | 0.343483 | 0.042773 |
| chr4_141516835_141522392_- | Spen | -1.60531 | 0.328664 | 0.037681 |
| chr3_118897093_118944335_+ | Dpyd | -1.78583 | 0.290009 | 0.019565 |
| chr11_117290397_117291036_+ | Sept9 | -1.84659 | 0.278048 | 0.021496 |
| chr14_31618971_31623096_- | Hacl1 | -2.23155 | 0.212929 | 0.03457 |
| chr6_31166475_31197851_- | AB041803 | -2.39632 | 0.189948 | 0.012109 |
| chr7_126551974_126552338_- | Eif3c | -2.43599 | 0.184796 | 0.016087 |
| chr3_118917072_118944335_+ | Dpyd | -2.47352 | 0.180052 | 0.014496 |
| chr18_74569766_74580534_+ | Myo5b | -2.51799 | 0.174586 | 0.019853 |
| chr9_59426122_59454469_- | Arih1 | -2.57476 | 0.167849 | 0.025746 |
| chr6_99429209_99439442_- | Foxp1 | -2.62246 | 0.16239 | 0.049601 |
| chr4_140562713_140570402_- | Arhgef10l | -2.64428 | 0.159953 | 0.016902 |
| chr19_40160554_40167623_- | Cyp2c70 | -2.67508 | 0.156575 | 0.006217 |
| chr5_28358273_28362234_+ | Rbm33 | -2.80764 | 0.142829 | 0.048968 |
| chr5_98003226_98004940_- | Antxr2 | -2.83827 | 0.139828 | 0.023423 |
| chr5_140105514_140132825_- | Mad1l1 | -3.04174 | 0.121436 | 0.001985 |
| chr19_41281870_41287625_- | Pik3ap1 | -3.24924 | 0.105168 | 0.023568 |
| chr10_87528298_87538325_+ | Pah | -3.31487 | 0.100491 | 0.049655 |
| chr8_70251613_70252101_+ | Sugp2 | -3.42209 | 0.093293 | 0.049535 |
| chr1_190871591_190888764_- | Rps6kc1 | -3.54104 | 0.085909 | 0.039349 |
| chr6_37353556_37364143_- | Creb3l2 | -3.69168 | 0.077392 | 0.016357 |
| chr15_85365276_85376702_+ | Atxn10 | -4.54455 | 0.04285 | 0.04303 |
| chr5_74536800_74542112_+ | Fip1l1 | -4.54455 | 0.04285 | 0.04303 |
| chr13_94528164_94532066_+ | Ap3b1 | -4.6913 | 0.038706 | 0.032299 |
| chr16_11413225_11420783_+ | Snx29 | -4.82818 | 0.035202 | 0.02464 |
| chr2_140030600_140057499_- | Tasp1 | -4.82818 | 0.035202 | 0.02464 |
| chr9_21742186_21742796_+ | Ldlr | -4.92468 | 0.032925 | 0.024757 |
| chr4_104845894_104865547_- | C8a | -4.94038 | 0.032569 | 0.025045 |
| chr11_101295034_101299076_- | Becn1 | -4.96493 | 0.032019 | 0.019066 |
| chr7_82671604_82674582_+ | Eftud1 | -4.9995 | 0.031261 | 0.019031 |
| chr8_83990427_83990985_+ | Prkaca | -5.21441 | 0.026934 | 0.01064 |
| chr11_16687936_16699485_- | not_annotated | -5.53297 | 0.021598 | 0.005366 |
