# Supplementary figures and images for: Circular RNA expression profiles and features in NAFLD mice: a study using RNA-seq data
Source: J Transl Med. 2020 Dec 11;18:476. doi: 10.1186/s12967-020-02637-w (PMC7731504; doi:10.1186/s12967-020-02637-w)

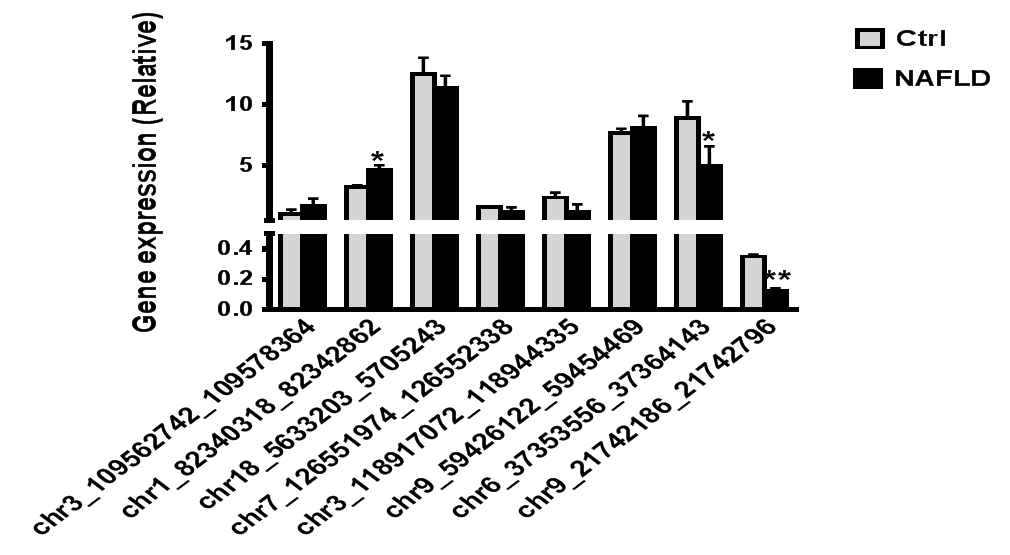

Supplement: Supplementary file 2 — Additional file 2: Figure S1. The expression levels of another eight random selected circRNAs after RT-qPCR validation. *p<0.05, **p<0.01. [file 12967_2020_2637_MOESM2_ESM.tif]
