# Supplementary material for: Circular RNA expression profiles and features in NAFLD mice: a study using RNA-seq data
Source: J Transl Med. 2020 Dec 11;18:476. doi: 10.1186/s12967-020-02637-w (PMC7731504; doi:10.1186/s12967-020-02637-w)
Supplement: Supplementary file 3 — Additional file 3: Table S2. Primers sequences of randomly selected eight circRNAs and two genes for another reverse transcription-quantitative polymerase chain-reaction (RT-qPCR). [file 12967_2020_2637_MOESM3_ESM.doc]

**Supplementary Table 2.** Primers sequences of randomly selected eight circRNAs and two genes for another reverse transcription-quantitative polymerase chain-reaction (RT-qPCR).

| **Forward** | **SeqTence (5'-3')** | **Reverse** | **SeqTence (5'-3')** | **Length** |
| --- | --- | --- | --- | --- |
| chr3_109562742_109578364-F1 | 5'-ATGTCACCCGAGTGAAGGTG-3' | chr3_109562742_109578364-R1 | 5'-GGTGGACCCAGATCCAACAT-3' | 85bp |
| chr1_82340318_82342862-F2 | 5'-CGGAGAACTGCACCCTGG-3' | chr1_82340318_82342862-R2 | 5'-TAGCAACCATTACTGCCCCG-3' | 121bp |
| chr18_5633203_5705243-F2 | 5'-GGCTTCAGATGCGAATTTTGG-3' | chr18_5633203_5705243-R2 | 5'-GCCAGCAGACCAGACAGTATTA-3' | 126bp |
| chr7_126551974_12655233-F1 | 5'-AGCCCCTGCTAGATCCTGTA-4' | chr7_126551974_12655233-R1 | 5'-TAGAAGCTCCTTGGCACGAC-4' | 132bp |
| chr3_118917072_118944335-F1 | 5'-CGAGCTCCATCTGTATGTGCT-3' | chr3_118917072_118944335-R1 | 5'-CAGGTGGTGATGTTGTGGGT-3' | 102bp |
| chr9_59426122_59454469-F1 | 5'-TCCTGCGCATGGTTGTGATA-3' | chr9_59426122_59454469-R1 | 5'-TCCCAATTGAAGTGGCTAAGGA-3' | 92bp |
| chr6_37353556_37364143-F1 | 5'-ACACCAATAGAGGAGGTGGAGA-3' | chr6_37353556_37364143-R1 | 5'-GCTGTGATGGTCAGAGTGACA-3' | 134bp |
| chr9_21742186_21742796-F1  mDDAH1-F  mVAV3-F | 5'-TGGCCATCTATGAGCTTCATGT-3'  5'-TGGCCATCTATGAGCTTCATGT-4'  5'- GCAACAGCTTGTTGAGTCCC-5' | chr9_21742186_21742796-R1  mDDAH1-R  mVAV3-R | 5'-GCCACTGGATGTTTTCGGTC-3'  5'-GCCACTGGATGTTTTCGGTC-3'  5'-AGCTCCCGCATATCTCTTGC-3' | 115bp  115bp  79bp |
