# Supplementary material for: Circular RNA expression profiles and features in NAFLD mice: a study using RNA-seq data
Source: J Transl Med. 2020 Dec 11;18:476. doi: 10.1186/s12967-020-02637-w (PMC7731504; doi:10.1186/s12967-020-02637-w)
Supplement: Supplementary file 4 — Additional file 4: Table S3. The circRNAs-miRNAs interaction network of top 300 dysregulated circRNAs. [file 12967_2020_2637_MOESM4_ESM.doc]

**Supplementary Table 3.** The circRNAs-miRNAs interaction network of top 300 dysregulated circRNAs.

| miRNA | circRNA |
| --- | --- |
| mmu-miR-6970-5p | chr1_121319596_121319972_- |
| mmu-miR-6401 | chr1_121319596_121319972_- |
| mmu-miR-33-3p | chr1_121319596_121319972_- |
| mmu-miR-149-5p | chr1_187225502_187233826_+ |
| mmu-miR-7087-3p | chr1_187225502_187233826_+ |
| mmu-miR-7032-5p | chr1_187225502_187233826_+ |
| mmu-miR-141-5p | chr1_187225502_187233826_+ |
| mmu-miR-6919-3p | chr1_187225502_187233826_+ |
| mmu-miR-3058-3p | chr1_187225502_187233826_+ |
| mmu-miR-7024-3p | chr1_187225502_187233826_+ |
| mmu-miR-1903 | chr1_187225502_187233826_+ |
| mmu-miR-3104-3p | chr1_187225502_187233826_+ |
| mmu-miR-320-5p | chr1_187225502_187233826_+ |
| mmu-miR-1188-3p | chr1_187225502_187233826_+ |
| mmu-miR-7008-3p | chr1_187225502_187233826_+ |
| mmu-miR-673-3p | chr1_187225502_187233826_+ |
| mmu-miR-6922-3p | chr1_187225502_187233826_+ |
| mmu-miR-7578 | chr1_187225502_187233826_+ |
| mmu-miR-5627-3p | chr1_187225502_187233826_+ |
| mmu-miR-7061-3p | chr1_187225502_187233826_+ |
| mmu-miR-6941-5p | chr1_190871591_190888764_- |
| mmu-miR-370-3p | chr1_190871591_190888764_- |
| mmu-miR-761 | chr1_190871591_190888764_- |
| mmu-miR-212-5p | chr1_190871591_190888764_- |
| mmu-miR-7074-5p | chr1_190871591_190888764_- |
| mmu-miR-883a-3p | chr1_190871591_190888764_- |
| mmu-miR-7668-3p | chr1_82340318_82342862_+ |
| mmu-miR-149-3p | chr1_82340318_82342862_+ |
| mmu-miR-7080-5p | chr1_82340318_82342862_+ |
| mmu-miR-7067-5p | chr1_82340318_82342862_+ |
| mmu-miR-5110 | chr1_82340318_82342862_+ |
| mmu-miR-7016-5p | chr1_82340318_82342862_+ |
| mmu-miR-7004-5p | chr1_82340318_82342862_+ |
| mmu-miR-504-3p | chr1_82340318_82342862_+ |
| mmu-miR-8091 | chr1_82340318_82342862_+ |
| mmu-miR-6978-3p | chr1_82340318_82342862_+ |
| mmu-miR-30c-1-3p | chr1_82340318_82342862_+ |
| mmu-miR-7047-5p | chr1_82340318_82342862_+ |
| mmu-miR-3102-5p.2-5p | chr1_82340318_82342862_+ |
| mmu-miR-196a-2-3p | chr1_82340318_82342862_+ |
| mmu-miR-6963-5p | chr10_107023653_107053516_- |
| mmu-miR-6963-5p | chr10_107045212_107053516_- |
| mmu-miR-370-3p | chr10_18089447_18096336_+ |
| mmu-miR-7667-3p | chr10_18089447_18096336_+ |
| mmu-miR-1231-5p | chr10_18089447_18096336_+ |
| mmu-miR-7003-5p | chr10_18089447_18096336_+ |
| mmu-miR-5114 | chr10_18089447_18096336_+ |
| mmu-miR-92a-2-5p | chr10_18089447_18096336_+ |
| mmu-miR-7081-5p | chr10_18089447_18096336_+ |
| mmu-miR-6934-5p | chr10_18089447_18096336_+ |
| mmu-miR-6964-5p | chr10_18089447_18096336_+ |
| mmu-miR-344-5p | chr10_18089447_18096336_+ |
| mmu-miR-30b-3p | chr10_18089447_18096336_+ |
| mmu-miR-7048-5p | chr10_18089447_18096336_+ |
| mmu-miR-6911-5p | chr10_18089447_18096336_+ |
| mmu-miR-6965-5p | chr10_18089447_18096336_+ |
| mmu-miR-6935-5p | chr10_18089447_18096336_+ |
| mmu-miR-6975-5p | chr10_18089447_18096336_+ |
| mmu-miR-6945-5p | chr10_18089447_18096336_+ |
| mmu-miR-344d-3-5p | chr10_18089447_18096336_+ |
| mmu-miR-7222-3p | chr10_87528298_87538325_+ |
| mmu-miR-207 | chr11_101295034_101299076_- |
| mmu-miR-337-3p | chr11_101295034_101299076_- |
| mmu-miR-6947-3p | chr11_101295034_101299076_- |
| mmu-miR-7073-3p | chr11_101295034_101299076_- |
| mmu-miR-6963-3p | chr11_101295034_101299076_- |
| mmu-miR-7036b-3p | chr11_101295034_101299076_- |
| mmu-miR-5625-3p | chr11_101295034_101299076_- |
| mmu-miR-5124a | chr11_101295034_101299076_- |
| mmu-miR-6911-5p | chr11_101295034_101299076_- |
| mmu-miR-7211-5p | chr11_106236994_106240099_+ |
| mmu-miR-6340 | chr11_116160061_116163359_- |
| mmu-miR-212-5p | chr11_117290397_117291036_+ |
| mmu-miR-665-5p | chr11_117290397_117291036_+ |
| mmu-miR-1952 | chr11_117290397_117291036_+ |
| mmu-miR-6410 | chr11_117290397_117291036_+ |
| mmu-miR-7023-5p | chr11_117290397_117291036_+ |
| mmu-miR-298-5p | chr11_117290397_117291036_+ |
| mmu-miR-432 | chr11_117290397_117291036_+ |
| mmu-miR-5620-5p | chr11_117290397_117291036_+ |
| mmu-miR-6955-5p | chr11_117290397_117291036_+ |
| mmu-miR-7069-5p | chr11_117290397_117291036_+ |
| mmu-miR-666-5p | chr11_117290397_117291036_+ |
| mmu-miR-6361 | chr11_117290397_117291036_+ |
| mmu-miR-3064-5p | chr11_117290397_117291036_+ |
| mmu-miR-149-5p | chr11_117290397_117291036_+ |
| mmu-miR-6914-5p | chr11_117290397_117291036_+ |
| mmu-miR-6971-5p | chr11_117290397_117291036_+ |
| mmu-miR-326-5p | chr11_117290397_117291036_+ |
| mmu-miR-6965-5p | chr11_72645536_72663173_+ |
| mmu-miR-1231-5p | chr11_72645536_72663173_+ |
| mmu-miR-7047-5p | chr11_72645536_72663173_+ |
| mmu-miR-6945-3p | chr11_96322110_96323445_+ |
| mmu-miR-7063-5p | chr12_4630636_4633562_+ |
| mmu-miR-1249-5p | chr12_4630636_4633562_+ |
| mmu-miR-7031-5p | chr12_4630636_4633562_+ |
| mmu-miR-29b-2-5p | chr13_3556313_3557057_+ |
| mmu-miR-207 | chr13_4507680_4514428_- |
| mmu-miR-344e-5p | chr13_4507680_4514428_- |
| mmu-miR-344h-5p | chr13_4507680_4514428_- |
| mmu-miR-7091-3p | chr13_4507680_4514428_- |
| mmu-miR-7214-5p | chr13_4507680_4514428_- |
| mmu-miR-6368 | chr13_94528164_94532066_+ |
| mmu-miR-3547-5p | chr13_94528164_94532066_+ |
| mmu-miR-363-3p | chr14_31618971_31623096_- |
| mmu-miR-1251-3p | chr14_78982235_78995163_+ |
| mmu-miR-351-5p | chr14_78982235_78995163_+ |
| mmu-miR-1946a | chr14_78982235_78995163_+ |
| mmu-miR-7659-5p | chr14_78994399_79026077_+ |
| mmu-miR-351-5p | chr14_78994399_79026077_+ |
| mmu-miR-6944-5p | chr14_78994399_79026077_+ |
| mmu-miR-6988-5p | chr14_78994399_79026077_+ |
| mmu-miR-3113-3p | chr15_77132716_77231437_- |
| mmu-miR-7020-5p | chr15_77132716_77231437_- |
| mmu-miR-3076-5p | chr16_11413225_11420783_+ |
| mmu-miR-3089-3p | chr16_11413225_11420783_+ |
| mmu-miR-680 | chr16_11413225_11420783_+ |
| mmu-miR-7064-5p | chr16_20656542_20657609_+ |
| mmu-miR-7003-5p | chr16_70433544_70441319_+ |
| mmu-miR-1903 | chr17_24992269_25003405_- |
| mmu-miR-6982-5p | chr17_24992269_25003405_- |
| mmu-miR-5113 | chr17_24992269_25003405_- |
| mmu-miR-665-3p | chr17_26083575_26084135_- |
| mmu-miR-7025-5p | chr17_64710703_64714712_+ |
| mmu-miR-6930-3p | chr17_66049785_66053091_- |
| mmu-miR-3105-5p | chr17_66049785_66053091_- |
| mmu-miR-497b | chr18_12684836_12698059_+ |
| mmu-miR-6947-3p | chr18_12684836_12698059_+ |
| mmu-miR-5627-3p | chr18_12684836_12698059_+ |
| mmu-miR-140-3p | chr18_12684836_12698059_+ |
| mmu-miR-429-3p | chr18_5633203_5705243_+ |
| mmu-miR-1190 | chr18_70580652_70593340_+ |
| mmu-miR-23a-5p | chr18_74569766_74580534_+ |
| mmu-miR-6537-3p | chr19_21678352_21684305_+ |
| mmu-miR-194-5p | chr19_21678352_21684305_+ |
| mmu-miR-6908-3p | chr19_21678352_21684305_+ |
| mmu-miR-1941-5p | chr19_40160554_40167623_- |
| mmu-miR-3100-5p | chr19_40160554_40167623_- |
| mmu-miR-7081-5p | chr19_41281870_41287625_- |
| mmu-miR-5110 | chr19_41281870_41287625_- |
| mmu-miR-696 | chr19_41281870_41287625_- |
| mmu-miR-7019-5p | chr19_41281870_41287625_- |
| mmu-miR-3544-3p | chr19_45630864_45636399_- |
| mmu-miR-667-5p | chr19_45630864_45636399_- |
| mmu-miR-6947-3p | chr19_55340713_55391868_+ |
| mmu-miR-207 | chr2_140030600_140057499_- |
| mmu-miR-1907 | chr2_140030600_140057499_- |
| mmu-miR-497a-5p | chr2_140030600_140057499_- |
| mmu-miR-1906 | chr2_140030600_140057499_- |
| mmu-miR-199a-3p | chr2_158492363_158493013_+ |
| mmu-miR-199b-3p | chr2_158492363_158493013_+ |
| mmu-miR-7035-5p | chr3_41581194_41596826_+ |
| mmu-miR-673-5p | chr4_104845894_104865547_- |
| mmu-miR-1930-5p | chr4_104845894_104865547_- |
| mmu-miR-3097-5p | chr4_104845894_104865547_- |
| mmu-miR-6958-3p | chr4_140562713_140570402_- |
| mmu-miR-7081-5p | chr4_140562713_140570402_- |
| mmu-miR-1906 | chr4_140562713_140570402_- |
| mmu-miR-18a-3p | chr4_140562713_140570402_- |
| mmu-miR-106a-3p | chr4_140562713_140570402_- |
| mmu-miR-7242-5p | chr4_140562713_140570402_- |
| mmu-miR-770-5p | chr4_140562713_140570402_- |
| mmu-miR-7665-3p | chr4_140562713_140570402_- |
| mmu-miR-6934-3p | chr4_140562713_140570402_- |
| mmu-miR-6914-5p | chr4_140562713_140570402_- |
| mmu-miR-7222-5p | chr4_140562713_140570402_- |
| mmu-miR-6947-3p | chr4_140562713_140570402_- |
| mmu-miR-17-3p | chr4_140562713_140570402_- |
| mmu-miR-20b-3p | chr4_140562713_140570402_- |
| mmu-miR-7074-5p | chr4_140562713_140570402_- |
| mmu-miR-7686-5p | chr4_140562713_140570402_- |
| mmu-miR-7658-3p | chr4_140562713_140570402_- |
| mmu-miR-6945-5p | chr4_140562713_140570402_- |
| mmu-miR-6990-3p | chr4_140562713_140570402_- |
| mmu-miR-7119-5p | chr4_141516835_141522392_- |
| mmu-miR-7024-5p | chr4_141516835_141522392_- |
| mmu-miR-7023-5p | chr4_141516835_141522392_- |
| mmu-miR-6941-5p | chr4_141516835_141522392_- |
| mmu-miR-7115-5p | chr4_141516835_141522392_- |
| mmu-miR-6971-3p | chr4_141516835_141522392_- |
| mmu-miR-449b | chr4_141516835_141522392_- |
| mmu-miR-1231-5p | chr4_141516835_141522392_- |
| mmu-miR-7074-3p | chr4_141516835_141522392_- |
| mmu-miR-6950-3p | chr4_141516835_141522392_- |
| mmu-miR-7211-3p | chr4_141516835_141522392_- |
| mmu-miR-7029-5p | chr4_141516835_141522392_- |
| mmu-miR-8103 | chr4_141516835_141522392_- |
| mmu-miR-6925-5p | chr4_46734057_46749582_- |
| mmu-miR-7054-5p | chr4_46734057_46749582_- |
| mmu-miR-7011-5p | chr4_46734057_46749582_- |
| mmu-miR-6912-5p | chr4_6822904_6842426_- |
| mmu-miR-149-3p | chr4_6822904_6842426_- |
| mmu-miR-7649-3p | chr5_140105514_140132825_- |
| mmu-miR-6919-3p | chr5_140105514_140132825_- |
| mmu-miR-7685-5p | chr5_140105514_140132825_- |
| mmu-miR-5110 | chr5_28358273_28362234_+ |
| mmu-miR-7030-5p | chr5_28358273_28362234_+ |
| mmu-miR-7075-5p | chr5_28358273_28362234_+ |
| mmu-miR-7076-5p | chr5_28358273_28362234_+ |
| mmu-miR-6980-5p | chr5_28358273_28362234_+ |
| mmu-miR-7023-5p | chr5_28358273_28362234_+ |
| mmu-miR-7081-5p | chr5_28358273_28362234_+ |
| mmu-miR-7056-5p | chr5_28358273_28362234_+ |
| mmu-miR-7044-5p | chr5_28358273_28362234_+ |
| mmu-miR-7024-5p | chr5_28358273_28362234_+ |
| mmu-miR-1968-5p | chr5_28358273_28362234_+ |
| mmu-miR-432 | chr5_28358273_28362234_+ |
| mmu-miR-6982-5p | chr5_28358273_28362234_+ |
| mmu-miR-1962 | chr5_28358273_28362234_+ |
| mmu-miR-6971-5p | chr5_28358273_28362234_+ |
| mmu-miR-6981-5p | chr5_28358273_28362234_+ |
| mmu-miR-7070-5p | chr5_28358273_28362234_+ |
| mmu-miR-667-5p | chr5_28358273_28362234_+ |
| mmu-miR-3078-3p | chr5_28358273_28362234_+ |
| mmu-miR-6914-5p | chr5_28358273_28362234_+ |
| mmu-miR-6910-5p | chr5_28358273_28362234_+ |
| mmu-miR-370-3p | chr5_28358273_28362234_+ |
| mmu-miR-6931-5p | chr5_28358273_28362234_+ |
| mmu-miR-6769b-5p | chr5_28358273_28362234_+ |
| mmu-miR-5623-3p | chr5_74536800_74542112_+ |
| mmu-miR-598-3p | chr5_74536800_74542112_+ |
| mmu-miR-486a-5p | chr6_119824385_119825819_- |
| mmu-miR-486b-5p | chr6_119824385_119825819_- |
| mmu-let-7a-5p | chr6_119824385_119825819_- |
| mmu-let-7e-5p | chr6_119824385_119825819_- |
| mmu-let-7b-5p | chr6_119824385_119825819_- |
| mmu-miR-7088-5p | chr6_119824385_119825819_- |
| mmu-miR-1843a-3p | chr6_119824385_119825819_- |
| mmu-miR-1249-5p | chr6_119824385_119825819_- |
| mmu-miR-125a-3p | chr6_141648488_141657606_+ |
| mmu-miR-1967 | chr6_141663577_141672313_+ |
| mmu-miR-669h-5p | chr6_3545509_3555466_+ |
| mmu-miR-7239-3p | chr6_37353556_37364143_- |
| mmu-miR-1903 | chr6_37353556_37364143_- |
| mmu-miR-7058-5p | chr6_37353556_37364143_- |
| mmu-miR-7049-5p | chr6_37353556_37364143_- |
| mmu-miR-7012-5p | chr6_37353556_37364143_- |
| mmu-miR-6974-3p | chr6_37353556_37364143_- |
| mmu-miR-1952 | chr6_37353556_37364143_- |
| mmu-miR-6915-5p | chr6_37353556_37364143_- |
| mmu-miR-7578 | chr6_37353556_37364143_- |
| mmu-miR-3620-5p | chr6_37353556_37364143_- |
| mmu-miR-298-5p | chr6_37353556_37364143_- |
| mmu-miR-6927-5p | chr6_37353556_37364143_- |
| mmu-miR-6954-5p | chr6_37353556_37364143_- |
| mmu-miR-7028-5p | chr6_37353556_37364143_- |
| mmu-miR-7044-5p | chr6_37353556_37364143_- |
| mmu-miR-7070-5p | chr6_37353556_37364143_- |
| mmu-miR-7001-5p | chr6_37353556_37364143_- |
| mmu-miR-877-3p | chr6_37353556_37364143_- |
| mmu-miR-7033-5p | chr7_126551974_126552338_- |
| mmu-miR-7663-5p | chr7_126551974_126552338_- |
| mmu-miR-485-3p | chr7_82671604_82674582_+ |
| mmu-miR-1929-5p | chr7_82671604_82674582_+ |
| mmu-miR-718 | chr8_13136795_13142598_+ |
| mmu-miR-6918-5p | chr8_70251613_70252101_+ |
| mmu-miR-6923-5p | chr8_70251613_70252101_+ |
| mmu-miR-7001-5p | chr8_70251613_70252101_+ |
| mmu-miR-6905-5p | chr8_70251613_70252101_+ |
| mmu-miR-1224-5p | chr8_70251613_70252101_+ |
| mmu-miR-7033-5p | chr8_70251613_70252101_+ |
| mmu-miR-6337 | chr8_70251613_70252101_+ |
| mmu-miR-7012-5p | chr8_70251613_70252101_+ |
| mmu-miR-760-5p | chr8_70251613_70252101_+ |
| mmu-miR-7034-3p | chr8_70251613_70252101_+ |
| mmu-miR-6975-5p | chr8_70251613_70252101_+ |
| mmu-miR-344g-3p | chr8_70251613_70252101_+ |
| mmu-miR-6998-5p | chr8_70251613_70252101_+ |
| mmu-miR-7117-5p | chr8_70251613_70252101_+ |
| mmu-miR-7000-5p | chr8_70251613_70252101_+ |
| mmu-miR-615-5p | chr8_83990427_83990985_+ |
| mmu-miR-7072-3p | chr8_84860905_84861430_+ |
| mmu-miR-3113-3p | chr8_94831772_94832700_- |
| mmu-miR-6357 | chr8_94831772_94832700_- |
| mmu-miR-3113-5p | chr8_94831772_94832700_- |
| mmu-miR-7032-5p | chr9_21569454_21575518_+ |
| mmu-miR-8095 | chr9_21569454_21575518_+ |
| mmu-miR-1946a | chr9_21569454_21575518_+ |
| mmu-miR-1946b | chr9_21569454_21575518_+ |
| mmu-miR-7019-5p | chr9_21569454_21575518_+ |
| mmu-miR-6964-5p | chr9_21569454_21575518_+ |
| mmu-miR-7212-3p | chr9_21569454_21575518_+ |
| mmu-miR-7037-5p | chr9_21742186_21742796_+ |
| mmu-miR-6919-5p | chr9_21742186_21742796_+ |
| mmu-miR-7001-5p | chr9_43005543_43008111_- |
| mmu-miR-346-5p | chr9_59426122_59454469_- |
| mmu-miR-7072-3p | chr9_59426122_59454469_- |
| mmu-miR-346-3p | chr9_86372340_86440754_- |
| mmu-miR-7001-5p | chr9_86372340_86440754_- |
| mmu-miR-298-5p | chr9_86372340_86440754_- |
| mmu-miR-7067-5p | chr9_86372340_86440754_- |
| mmu-miR-6905-5p | chr9_86372340_86440754_- |
| mmu-miR-365-2-5p | chr9_86372340_86440754_- |
| mmu-miR-1231-5p | chr9_86372340_86440754_- |
| mmu-miR-296-3p | chr9_86372340_86440754_- |
| mmu-miR-1946b | chr9_86372340_86440754_- |
| mmu-miR-709 | chr9_86372340_86440754_- |
| mmu-miR-5615-5p | chr9_86372340_86440754_- |
| mmu-miR-6953-5p | chr9_86372340_86440754_- |
| mmu-miR-7010-3p | chrX_56643367_56655053_+ |
